# miRNAs in the Era of Personalized Medicine: From Biomarkers to Therapeutics

**DOI:** 10.3390/ijms22158154

**Published:** 2021-07-29

**Authors:** Bárbara A. Mc Cormack, Eva González-Cantó, Cristina Agababyan, Nancy A. Espinoza-Sánchez, Sarai Tomás-Pérez, Antoni Llueca, Josep Marí-Alexandre, Martin Götte, Juan Gilabert-Estellés

**Affiliations:** 1 Research Laboratory in Biomarkers in Reproduction, Gynaecology and Obstetrics, Research Foundation of the General University Hospital of Valencia, 46014 Valencia, Spain; barbymccormack@gmail.com (B.A.M.C.); evagonzalezcanto@gmail.com (E.G.-C.); dra.kristina.agababyan@gmail.com (C.A.); sarai.altea@gmail.com (S.T.-P.); juangilaeste@yahoo.es (J.G.-E.); 2 Obstetrics and Gynaecology Service, General University Hospital of Valencia Consortium, 46014 Valencia, Spain; 3 Research Laboratory, Department of Gynecology and Obstetrics, Münster University Hospital, D-48149 Münster, Germany; nancyadriana.espinozasanchez@ukmuenster.es; 4 Department of Medicine, University Jaume I, 12071 Castellón, Spain; antonillueca@gmail.com; 5 Multidisciplinary Unit of Abdominal Pelvic Oncology Surgery (MUAPOS), General University Hospital of Castellón, 12004 Castellón, Spain; 6 Department of Paediatrics, Obstetrics and Gynaecology, University of Valencia, 46010 Valencia, Spain

**Keywords:** miRNAs, epigenetics, biomarker, therapeutics, personalized medicine

In recent years, interest in personalized medicine has considerably increased. As its name suggests, this area of medicine is based on finding and weighing the individualities of each patient. Part of the reason for this growth in interest is due to the improved understanding by the biomedical scientific community of the unique characteristics of each individual (at the molecular, physiological, environmental sensitivity, healthcare access, and lifestyle levels) and their influence on treatment response [1]. Besides, due to the clinical need for new diagnostic and monitoring alternatives that complement the current evaluations, the use of next-generation sequencing (NGS) technologies in the health field has been implemented. These techniques include the genotyping and sequencing of patients’ genomes, and characterization of epigenetic traits (e.g., genome methylation and miRNA expression). With regards to oncology, the technical and methodological advances have permitted the incorporation to the clinical scenario, for instance, of one-step nucleic acid amplification for CK19 characterization in the sentinel node in breast cancer [2], the Proactive Molecular Risk Classifier for Endometrial Cancer [3], and tumor sequencing to identify specific gene mutations to decide which treatments apply, as for example osimertinib in patients with lung cancer and *EGFR* mutations [4,5]. Although NGS implementation represents a challenge in terms of storage and interpretation of the massive amounts of produced data, it is undoubtedly improving our understanding and evaluation of the risk of diseases and early diagnosis for each patient [6].

The interest in offering a more effective health option has extended to scientific research groups, motivating them to investigate and identify different biomolecules capable of acting as biomarkers of different diseases. A biomarker considers every characteristic that is objectively measured and evaluated as an indication of either normal or pathogenic biologic processes, or pharmacologic responses to a therapeutic intervention [7]. In this context, non-coding RNAs have become a focal point of personalized medicine.

After the Human Genome Project, it became evident that there is an unexpectedly low percentage of protein-coding genes. Thus, the central dogma of the molecular biology of 1958 [8] was questioned. It is currently accepted that the high degree of human complexity is partly due to the intricate regulatory network of non-coding RNAs on physiological and pathophysiological processes. In this scenario, microRNAs (miRNAs) represent the most abundant class of small endogenous non-coding RNAs. They constitute one of the largest conserved gene families among species suggesting their important role in the regulation of different biological and developmental processes. In the majority of cases, such regulation occurs once these small molecules bind to the 3′ untranslated region (3′-UTR) of their target mRNA through Watson–Crick complementarity [9], however, other binding interactions such as interactions with the 5′-UTR or the coding region of mRNAs have been reported as well [10,11]. The complexity of this interaction lies in the fact that several miRNAs can target a specific mRNA, while a single miRNA can target several mRNAs [10,11]. They can act at the post-transcriptional level by either inhibiting the translation or promoting the degradation of their target mRNAs [10,12,13].

The expression of miRNAs has been widely studied, leading to the identification of 1917 precursors, and 2654 mature sequences as registered in the miRBase registry (available online at: http://www.mirbase.org) (release 22, accessed on 1 July 2021). Whilst most are ubiquitously expressed, there is a small tissue/cell-specific set that is often deregulated in diseases [14]. This characteristic, together with the discovery of their presence in different fluids (such as tear fluid, urine, breast milk, seminal fluid, saliva, amniotic fluid, bronchial lavage, cerebrospinal fluid, pleural fluid, peritoneal fluid, and colostrum) [15] confers their biomarker value. Moreover, circulating miRNAs are usually contained in vesicles, protein complexes, or lipoprotein complexes, which increase their stability and resistance to degradation by endogenous RNAse activity [16,17].

The detection of circulating miRNAs would improve personalized medicine since one of the current clinical challenges is to develop minimally invasive approaches. They show remarkable stability in bodily fluid and consistent expression profiles, having the potential to serve as biomarkers for changes in physiological and pathological conditions [14]. Undoubtedly, their detection in liquid biopsies would allow minimally invasive monitoring of patients, thus facilitating an optimal therapy selection. Liquid biopsies refer to the use of different types of biological fluids, obtained by non-invasive or minimally invasive techniques, as a medium in which to analyze a patient [18,19]. In patients with ovarian cancer, it is even possible to isolate circulating tumor DNA and cell-free DNA, whose sequencing allows disease monitoring [20]. In this context, the standardization of the sample collection and processing (preservation, manipulation, and miRNA detection) is crucial so that we can face a more accurate tool for patient management [17]. This would improve both the patient’s quality of life and the overall cost to the public health system.

Interesting examples in this area have been reported. For instance, Zampetaki et al. [16] stated that the expression signatures of circulating miRNAs are emerging as novel biomarkers of numerous diseases. Their research in the cardiovascular field postulates that it is likely that the specific circulating miRNA content reflects the specific activation state of circulating cells in a patient, as platelet-enriched miR-223 and mi-R197, and endothelial-cell and platelet-enriched miR-126 [21]. However, as reviewed by Schulte et al. [22], cardiovascular biomarkers are still protein’s domain. Given this evidence, circulating miRNAs information could provide an integrated readout of cellular activation and tissue injury in response to cardiovascular risk factors and disease that could potentially replace the current cardiovascular biomarkers based on proteins.

miRNA profiling in plasma from patients suffering from diabetes also revealed that non-coding RNAs have been implicated in the epigenetic regulation of key metabolic, inflammatory, and antiangiogenic pathways in type 2 diabetes. In particular, the loss of endothelial miR-126 might explain the impaired peripheral angiogenic signaling in patients [23]. In the current special issue, outstanding examples have been provided by Lin and Tsai [24] and Oto et al. [25] in bladder and renal cell cancer, respectively, and by Salloum-Asfar et al. in Autism Spectrum Disorder [26].

In oncology, it is well known that miRNAs are deregulated in tumor tissue, and this promotes cellular processes that allow malignant cells to survive. Pajares et al. [27] summarize recent findings on epigenetic regulation of miRNAs in cancer, including DNA methylation and histone modifications. For instance, deregulation of miR-200b, miR-200c, miR-205, and miR-487 has been observed in lung cancer [12], supporting the concept that smoking-related epigenetic changes might be considered as putative biomarkers for lung diseases. Moreover, the development of novel cost-effective extracellular vesicle-based liquid biopsy techniques aid miRNA-based diagnostics, as recently demonstrated for inflammatory breast cancer [28].

Regarding endometrial cancer, the most frequent gynecological malignancy, Widodo et al. [29] analyzed the role of twelve miRNAs in the endometrial carcinogenesis pathway. Their results indicated that three of them have different expressions between cancer and normal tissue. miR-495 and miR-152 were downregulated, whereas miR-181d was up-regulated in endometrial cancer compared to normal tissue. Other miRNA families that were also associated with this pathology were miRNA-200 and miRNA-30c. Likewise, these families were also studied in ovarian cancer, either as therapeutic targets or as an indicator of significantly better disease-free or overall survival, respectively [30].

Abnormal miRNA expression has also been described in non-malignant gynecological pathologies as endometriosis. Endometriosis is, by definition, a multifactorial and polygenic disease and recent research provided evidence that a wide variety of genes involved in essential systems for the pathophysiology of this disease are potentially regulated by miRNAs. As reviewed by Marí-Alexandre et al. [30] a large number of studies have described different patterns of miRNA expression between the eutopic endometrium of healthy women and patients, as well as in their ectopic lesions. Using a high-throughput miRNA sequencing approach, Saare et al. [31] identified five over-expressed miRNAs (miR-34c, -449a, -200a, -200b, -141) which makes it possible to discriminate peritoneal lesions from surrounding healthy tissue. An extensive list of deregulated miRNAs is detailed, from in vitro assays to assays performed on patient samples considering distinct biofluids [13].

Apart from endometriosis, a dysregulation of specific miRNAs is linked to a broad spectrum of diseases in women, as summarized for the example let-7d by De Santis and Götte [32]. Let-7d dysregulation contributes to the pathogenesis of female malignancies, endometriosis and pregnancy-associated diseases including preeclampsia and fetal growth restriction. From a technical point of view and regardless of the disease of study, it is undoubtably that the normalization process of RT-qPCR results might deeply influence the results. Therefore, investigations of the best normalizer are of great interest in the miRNA research flied, as illustrated by Oto et al [25].

The undoubted coupling of miRNAs and personalized medicine motivated this Special Issue. The studies accepted for this special issue cover a variety of aspects of the role of miRNAs in medicine research. A total of 5 original papers and 3 reviews have been published, as summarized in Table 1.

We would like to thank all the authors and research groups that have contributed to this special issue and the staff members of the International Journal of Molecular Science (IJMS) for their editorial support. These published articles are evidence of the growing interest in the field and illustrate advances in miRNA research. We hope it provides insights that help both researchers and healthcare specialists to continue on the way towards the ever-growing field of microRNAs in personalized medicine.

## Figures and Tables

**Table 1:** Contributors to the Special Issue.

Authors	Title	Topic	Type
Muñoz-Hidalgo et al. [33]	The Status of EGFR Modulates the Effect of miRNA-200c on ZEB1 Expression and Cell Migration in Glioblastoma Cells	Glioblastoma	Original Research
Lin and Sai [24]	Circulating miRNAs Act as Diagnostic Biomarkers for Bladder Cancer in Urine	Bladder cancer	Original Research
Liu et al. [34]	Canonical and Interior Circular RNAs Function as Competing Endogenous RNAs in Psoriatic Skin	Psoriasis	Original Research
Kinoshita et al. [35]	Interplay of RNA-binding proteins and microRNAs in neurodegenerative diseases	Neurogenerative diseases	Review
Salloum-Asfar et al. [26]	Circulating non-coding RNAs as a Signature of Autism Spectrum Disorder Symptomatology	Autism Spectrum	Original Research
De Santis and Götte [32]	The Role of microRNA Let-7d in Female Malignancies and Diseases of the Female Reproductive Tract	Female malignancies and reproduction	Review
Pajares et al. [27]	Epigenetic Regulation of microRNAs in Cancer: Shortening the Distance from Bench to Bedside	Cancer	Review
Oto et al. [25]	Identification of miR-20a-5p as robust normalizer for urine microRNA studies in Renal Cell Carcinoma and a profile of dysregulated microRNAs	Renal cell carcinoma	Original research
